# The Man and the Book

**Published:** 1890-07-12

**Authors:** 


### THE DOCTOR'S PULPIT.

### THE MAN AND THE BOOK.

THE man of the season is Stanley; the book of the season is Stanley's. This is an object lesson both for writers and readers. Writers unfortunately are not always the readiest to make use of object lessons; they are apt to think that because they can write they have passed entirely beyond the pupil stage. Having become teachers they have ceased to be learners. But the Kingdom of Heaven is not the only kingdom that requires a little child's heart of its subjects. The kingdom of science, of general knowledge, of literature, of practical wisdom and sense, all demand exactly the same child's docility of temper. That writer who despises object lessons, nay more who does not keep his eyes constantly open looking for them, will certainly not write long, for nobody will want to read him. He who gets all his lore from his book-shelves may succeed in putting his own lore on somebody else's book-shelves; but he will seldom have the happiness of putting it anywhere else. The industrious man of books may write, just as the mechanical pigeon may fly; but since the man has no more original vitality in him than the pigeon, he can never become immortal in an increasing host of living and believing successors. The mere literary filter has its uses; but it is only a filter when all is said, and not a living borage plant which may cool and purify the water for all future generations.

But the reading public is often as dull in perceiving the use of an object lesson as writers are in despising it. Readers find themselves much at sea in the market of books. They do not know how to value them. Instead of judging for themselves they wait for the judgment of the critics; and as the critics in many cases have no more skill in judgment than the readers, but only more assurance, their help is not seldom rather of the nature of a hindrance. It would be of great practical service to readers and book-buyers if they could be provided with some general standard of literary value. Stanley and his book point out such a standard; and it is seen to be one which is capable of universal application.

Why is Stanley himself, and why is his book so complete and striking a success? Why has the great traveller thrown an enchanter's net over the hearts and minds of men and women as different from each other and from him as are the variously-coloured flowers in a garden or the wild beasts that inhabit the primeval forest? It is not because of what he has said or sung: not because he is a novelist, a historian, or a poet. But it is because he has purposed, and carried out, a work requiring the highest courage day after day, the clearest and readiest intelligence day after day, the most arduous and toilsome exertions day after day, the most resolute and unflinching determination day after day, and practically all the highest qualities of which the human creature at its noblest is capable—it is because he has initiated and carried to an end a work so great that the concentrated energy of all humanity seemed to be required for it; it is for this reason that he himself as the doer of the work, and his book as the story of it, are in all men's mouths and minds and hearts.

There emerges from the story of Stanley and his book this very old, but much neglected, standard of literary value: a noble experience nobly told. The standard has been well tried and approved. The trials of it have not been confined to the men and women of the present generation. That is well; for the men and women of the present generation are for the most part very poor judges of literature. They have not the means of forming judgments. Their personal experience is altogether too narrow, too shallow, too poor in variety, too conventional, too much of the drawing-room and the newspaper office and the suburban villa; too unheroic, too little of the strong man resolutely grappling with a varying destiny, and too much of the man who commits suicide because his morning coat is shabby and he has not been able to afford a new swallowtail for five years.

There are certain books that have survived from the earliest ages, living on in spite of the most strange and adverse circumstances. The book of Job is an example. Why does it live? When will it die? It lives because it is a noble experience nobly told. It will die never, unless man should defy all the predictions of the divinest sages and all the conclusions of the evolutionist philosophers. "If a man die shall he live again?" The questioner who faced the unknown with such a bold though foreboding front lives yet in the souls of all who see the tragedies that he saw, and hope for the restitution that he hoped for. The intellectual and order-searching labours of Aristotle survive in the minds of all who hate confusion and anarchy, and rest not until they see the eternal law established in everything that lives and moves. So the dim gropings, alternating with the clear daylight progress, of Socrates, are immortal, because they are the story of a man whose very life it was to think the thoughts and do the deeds he did. Poor John Bunyan, in his Bedford jail! Who would have ventured to prophesy that there was literary immortality in him? Of his "Pilgrim's Progress" his humble friends and neighbours were not slow to express their opinions. "Some said, 'Print it, John:' others said 'No!'" What the literary critics of the time would have said had they been consulted it is not difficult to guess. They would have advised the tinker to stick to his soldering-iron.

What every reader should look for in his book and even in his newspaper is not cleverness, but originality. Of course a man's peculiar originality may be cleverness, and that kind of cleverness is sterling gold. But the cleverness of the trained literary acrobat or harlequin, though it may amuse an evening, has no staying power, and cannot supply either food or lodging to the mind, which is in need of both. Many people object that if they read nothing but what is original they will seldom read anything at all. To this it may be replied that they would probably be both stronger and clearer in mind than they are if they were to cut off nineteen-twentieths of their reading. The man of many books is very commonly the man of many feeblenesses. Moreover, originality does not consist in newness; for, notwithstanding all modern researches and discoveries, Solomon was very largely right when he said "There is nothing new under the sun." The real originality is when a man puts the whole of his soul into his work. It may not be much of a soul. It may not have a great deal either of light, or force, or finish, or loveliness about it. But such as it is, there it is, a new soul, different from, though like unto, every other soul that ever lived its life and told the story of it in a book.

Life is made up of thoughts, of passions, of purposes, of deeds. This is the only substance out of which true books can be made. This is the substance out of which Stanley's book is made; and so the book lives; and so it quickens life in others; and so it will continue to live, and to quicken life. Stanley may thus render to this generation a much greater service than even his deeds and discoveries in Africa. He may, nay he will, act as a powerful chalybeate poured into the bloodless arteries of the newspaper and book writers of the time. Great need is there of this impulse to vitality. Much of the writing of the day is like the pallid faces of its authors, and for the same reason. They live in a world bounded by wall-papers, and glass windows, and ceilings ten feet high. They exhaust the air in a few breaths; and then both freshness and vitality are gone. The practically unlimited atmosphere of the open air should be the life of the body at least ten or twelve hours in the twenty-four; and so should the still freer and fresher atmosphere of infinity be the life of the mind. Books that breathe nothing of the freshness and strength of the infinite are seldom worth reading, and are destined to an oblivion that will be final and complete.

